# H⁺ Exchange‐Driven ppb‐Level and High‐Selective Formaldehyde Detection at Room Temperature for Environmental and Clinical Applications

**DOI:** 10.1002/advs.202518324

**Published:** 2025-12-08

**Authors:** Lubing Cai, Mengyang Pang, Zhaosong Liu, Yanfei Li, Jiani Li, Zhaorui Zhang, Fengshuang Zheng, Chao Li, Ang Zheng, Xuemin Zhang

**Affiliations:** ^1^ Department of Chemistry College of Sciences Northeastern University Shenyang Liaoning 110819 China; ^2^ Department of Breast Surgery The First Hospital of China Medical University Shenyang Liaoning 110001 China; ^3^ State Key Laboratory of Digital Steel Northeastern University Shenyang Liaoning 110819 China; ^4^ School of Arts Northeastern University Shenyang Liaoning 110819 China; ^5^ State Key Laboratory of High‐performance Precision Manufacturing Dalian University of Technology Dalian 116024 China

**Keywords:** breath sensor, H^+^ exchange, high‐selectivity, indoor air quality monitoring, room temperature formaldehyde sensor

## Abstract

Formaldehyde is both a pervasive air pollutant and a critical breath biomarker for tumor‐related diseases, yet its reliable detection remains difficult due to ultralow concentrations and interference from ubiquitous volatile organic compounds (VOCs). Here, an H⁺‐exchange strategy is reported that markedly enhances the sensing performance of sodium titanate (Na_2_Ti_3_O_7_, NTO) by introducing abundant surface hydroxyl groups and tuning conduction pathways. In H⁺‐exchanged NTO (H‐NTO), hydroxyl groups act as selective adsorption sites for formaldehyde, while formaldehyde adsorption simultaneously suppresses surface‐proton and internal‐electron conduction by increasing the activation energy for proton hopping and generating electron‐trapping states. This dual modulation effectively eliminates cross‐sensitivity to other VOCs (e.g., methanol), enabling H‐NTO to achieve an ultralow detection limit of 2 ppb, a wide dynamic range up to 100 ppm, and stable operation over two months—contrasting with the negligible response of pristine NTO. To demonstrate practical utility, we developed a handheld H‐NTO prototype for wireless indoor air‐quality monitoring and non‐invasive breath‐based breast cancer screening. Coupled with machine learning, the system achieved high diagnostic accuracy, establishing H⁺‐exchange as a powerful route toward next‐generation intelligent formaldehyde sensors.

## Graphical abstract

This article presents an innovative room‐temperature formaldehyde sensor based on H⁺ exchange method, achieving ppb‐level detection limit and exceptional selectivity. The study highlights its practical potential for real‐time environmental monitoring and clinical diagnostic applications.

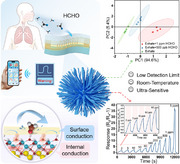

## 1 Introduction

The development of low‐cost and reliable gas sensors is crucial for advancing environmental monitoring and medical diagnostics, particularly in applications such as breath analysis.^[^ ^1^, ^2^, ^3^, ^4^ ^]^ Among available technologies, chemiresistive sensors are especially promising,^[^ ^5^, ^6^, ^7^ ^]^ yet most rely on metal oxide semiconductors (MOSs) such as TiO_2_, SnO_2_, and ZnO, which require elevated operating temperatures (200–400 °C) to activate surface redox chemistry with oxygen species (O_2_^−^, O^−^, O^2−^).^[^ ^8^, ^9^, ^10^, ^11^ ^]^ This thermal dependence limits their integration into portable, low‐power devices. Moreover, the intrinsic oxygen ionosorption mechanism often leads to poor selectivity and unsatisfactory limits of detection (LOD), preventing reliable operation at sub‐ppm concentrations and in complex environments such as exhaled breath. Consequently, achieving highly selective, ppb‐level gas detection under room‐temperature (RT) conditions remains a major challenge.

To overcome these limitations, alternative sensing platforms are being explored.^[^ ^12^, ^13^, ^14^ ^]^ Our recent work highlights sodium titanate (Na_2_Ti_3_O_7_, NTO), a 2D ion‐conducting material,^[^ ^15^, ^16^ ^]^ as a promising candidate for next‐generation chemiresistive sensors. Its selectivity can be enhanced through catalytic nanoparticle (NP) decoration—for example, Pd NP‐decorated NTO enables ppb‐level H_2_ detection, while AuAg NP‐decorated NTO shows exceptional selectivity toward CH_3_CN.^[^ ^17^, ^18^ ^]^ However, the fundamental sensing mechanism of titanates remains insufficiently understood. In particular, the role of counterions intrinsic to NTO (e.g., Na⁺, H⁺) has not been systematically investigated, despite their critical influence on ion transport and potential involvement in gas adsorption. For instance, incorporation of H⁺ promotes the formation of surface hydroxyl groups that may act as hydrogen‐bond donors for recognizing hydrogen‐bond acceptor molecules such as formaldehyde (HCHO). This implies that counterion engineering could be a powerful yet underexplored approach for tuning titanate‐based sensors.

Among volatile organic compounds (VOCs), formaldehyde is especially critical: while extensively applied in chemical manufacturing, agriculture, construction, and food preservation, it poses severe threats to human health.^[^ ^19^ ^]^ Chronic formaldehyde exposure is linked to respiratory and immune disorders,^[^ ^20^, ^21^ ^]^ leading the World Health Organization (WHO) to set a long‐term exposure threshold of 80 ppb.^[^ ^22^ ^]^ Furthermore, clinical studies report elevated formaldehyde concentrations in the exhaled breath of breast cancer patients, ranging from ≈500 ppb in Stage I to ≈1.2 ppm in Stage IV,^[^ ^23^, ^24^, ^25^ ^]^ highlighting formaldehyde's potential as a non‐invasive biomarker for early diagnosis.

Here, we introduce an H⁺‐exchange strategy to enable highly selective, ppb‐level detection of HCHO at RT using titanate materials. Specifically, sodium titanate was treated with dilute HCl to replace Na⁺ counterions with H⁺, yielding H⁺‐exchanged NTO (H‐NTO). Unlike pristine NTO, which shows negligible response, H‐NTO exhibits pronounced and selective HCHO sensing across a wide dynamic range (2 ppb–100 ppm). First‐principles calculations reveal that surface hydroxyl groups from H⁺ exchange provide preferential adsorption sites for HCHO via hydrogen bonding, while formaldehyde adsorption simultaneously suppresses both surface‐proton and internal‐electron conduction by increasing proton hopping barriers and inducing electron‐trapping states. These synergistic effects account for the sensor's ultralow LOD, high selectivity, and long‐term stability (>60 days).

To demonstrate practical utility, we integrated H‐NTO into a wireless handheld prototype for real‐time indoor air‐quality monitoring and applied it to non‐invasive breath analysis. The system triggered smartphone alerts when HCHO exceeded WHO safety limits and, when combined with a 1D convolutional neural network (1D‐CNN), discriminated between breath samples from healthy individuals and breast cancer patients with 99% accuracy. Collectively, this work establishes ion exchange as a powerful strategy for regulating gas adsorption and conduction in titanates, offering both mechanistic insights and practical design principles for next‐generation intelligent sensors targeting HCHO and beyond.

## 2 Results and Discussions

### 2.1 Characterization of NTO and H‐NTO

**Figure 1:**
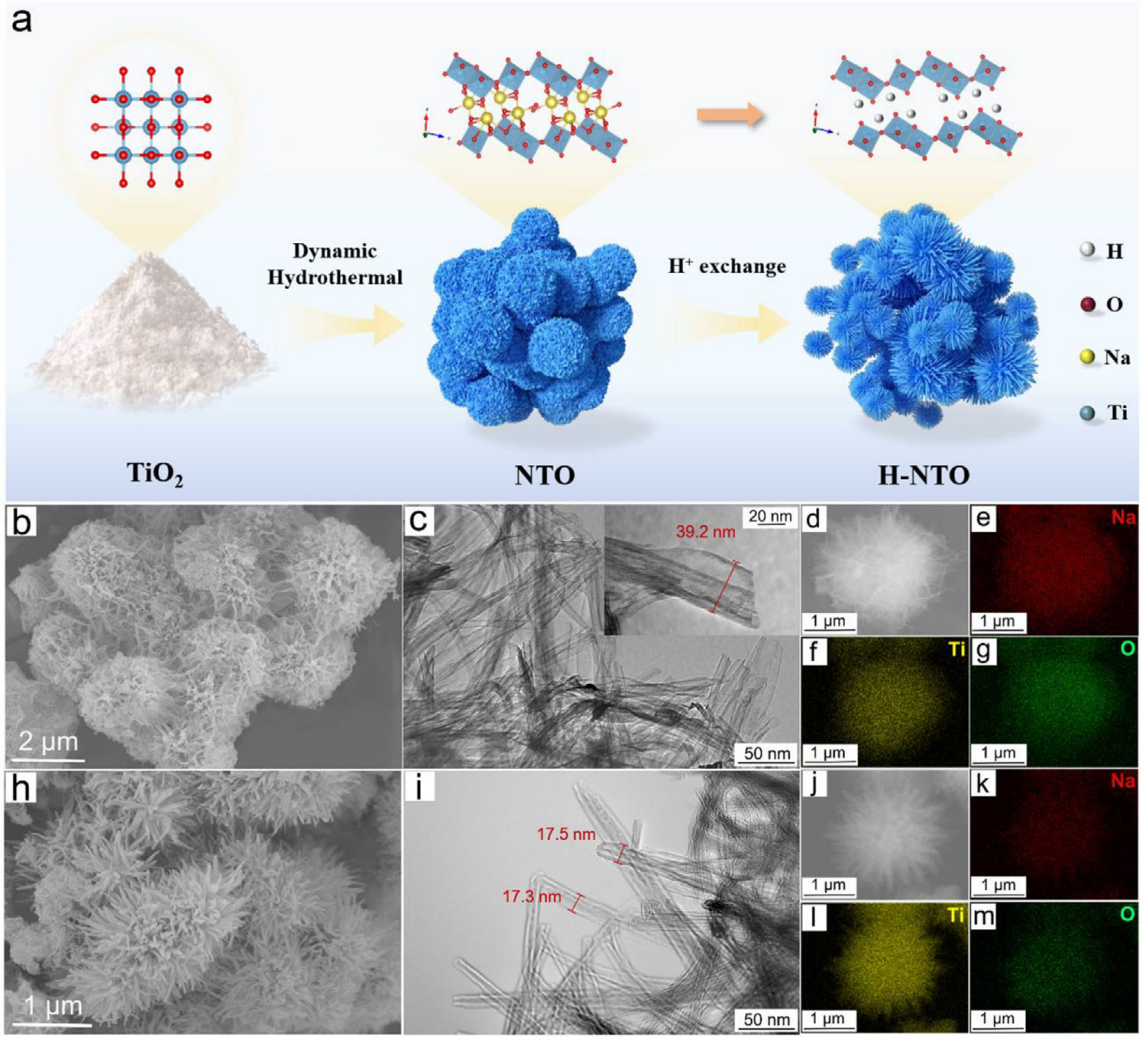
Structural characterization of NTO and H‐NTO. a) Schematic illustration of the preparation of H‐NTO via H⁺ exchange. b,c) SEM and TEM images of pristine NTO. d) EDX surface scanning profile of NTO, showing the distribution of e) Na, f) Ti, and g) O elements. h,i) SEM and TEM images of H‐NTO. j) EDX surface scanning profile of H‐NTO, showing the distribution of k) Na, l) Ti, and m) O elements.

A schematic illustration of the H‐NTO preparation process is shown in **Figure** **1**a. Pristine NTO was first synthesized via a hydrothermal method using TiO_2_ powder as the precursor.^[^ ^26^, ^27^ ^]^ Subsequently, ion exchange was carried out by dispersing NTO into dilute HCl solutions, yielding H‐NTO.^[^ ^28^, ^29^ ^]^ Specifically, H‐NTO C1, C2, C3, and C4 correspond to H‐NTO prepared with HCl concentrations of 10^−1^ m, 10^−2^ m, 10^−3^ m, and 10^−4^ m, respectively.

The morphology of the pristine and modified materials was characterized by scanning electron microscopy (SEM) and transmission electron microscopy (TEM) (Figure 1). Pristine NTO exhibits an interwoven, flower‐like architecture with a diameter of ≈2 µm, composed of stacked nanoribbons radiating outward, each ≈30–40 nm in width (Figure 1b,c). The H⁺ exchange process in 10^−3^ m HCl preferentially dissolves amorphous regions, thereby generating H‐NTO with more uniform and well‐defined structures (Figure 1h,i). At higher acid concentrations, however, the etching effect becomes dominant, leading to progressive degradation of the titanate framework (Figure S1, Supporting Information).

**Figure 2:**
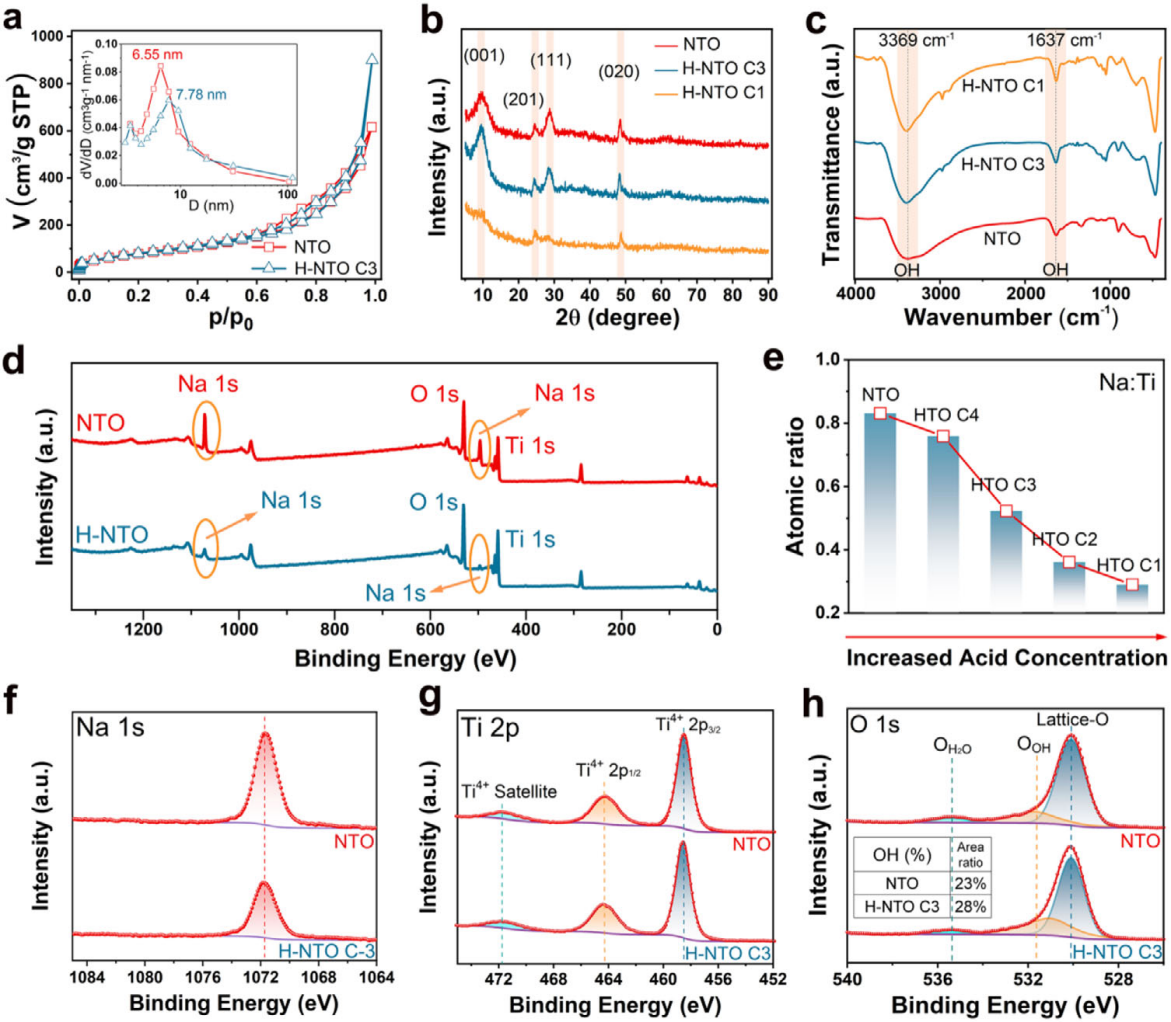
Structural and compositional analysis of NTO and H‐NTO. a) N_2_ adsorption‐desorption isotherms of NTO and H‐NTO C3, with BJH pore size distribution shown in the inset. b) XRD patterns. c) FTIR spectra. d) XPS survey spectra. e) Variation of Na: Ti atomic ratio with increasing acid concentration. High‐resolution XPS spectra of f) Na 1s, g) Ti 2p, and h) O 1s.

Brunauer‐Emmett‐Teller (BET) measurements indicate that the H⁺‐exchange process slightly increases the specific surface area of NTO (**Figure** **2**a). The X‐ray diffraction (XRD) patterns (Figure 2b) display characteristic peaks at 9.3°, 24.4°, 28.4°, and 48.6° for pristine NTO, consistent with the standard JCPDS card No. 31‐1329 for Na_2_Ti_3_O_7_ (Figure S2, Supporting Information).^[^ ^17^ ^]^ Upon H⁺ exchange, the diffraction peaks of H‐NTO C3 become sharper and more intense, reflecting improved crystallinity. Moreover, the (001) reflection gradually shifts toward higher angles, indicative of reduced interlayer spacing. By contrast, for H‐NTO C1, the diffraction peaks are barely discernible, a consequence of partial dissolution and structural collapse of NTO under highly concentrated HCl treatment.^[^ ^28^, ^30^ ^]^

The H⁺‐exchange process in NTO can be monitored through elemental and spectroscopic analyses. Energy‐dispersive X‐ray spectroscopy (EDX) mapping (Figure 1d–g) shows uniform distribution of Na, Ti, and O in pristine NTO, with a Na/Ti atomic ratio close to the stoichiometric value of Na_2_Ti_3_O_7_. Increasing the HCl concentration progressively decreases the Na/Ti ratio (Figure 2e), indicating partial substitution of Na⁺ by H⁺. Consistently, X‐ray photoelectron spectroscopy (XPS) reveals a marked decrease in Na 1s peak intensity (1070 and 494 eV) after H⁺ exchange (Figure 2d), and gradually weakens with increasing H^+^ concentration (Figure 2f; Figure S3, Supporting Information), confirming the successful substitution of Na⁺ by H⁺. Notably, the Ti 2p spectrum remains largely unchanged (Figure 2g), suggesting that the TiO_6_ framework is preserved. In contrast, the O 1s spectrum shows a significant increase in the hydroxyl oxygen peak (531 eV), indicative of abundant surface hydroxyl groups (Figure 2h). Fourier‐transform infrared spectroscopy (FTIR) further corroborates this observation: the intensities of the O─H stretching (3369 cm^−1^) and bending (1637 cm^−1^) vibrations are enhanced after H⁺ exchange, confirming the formation of a hydroxyl‐rich surface in H‐NTO.^[^ ^31^, ^32^ ^]^

### 2.2 HCHO Sensing Performance of H‐NTO

**Figure 3:**
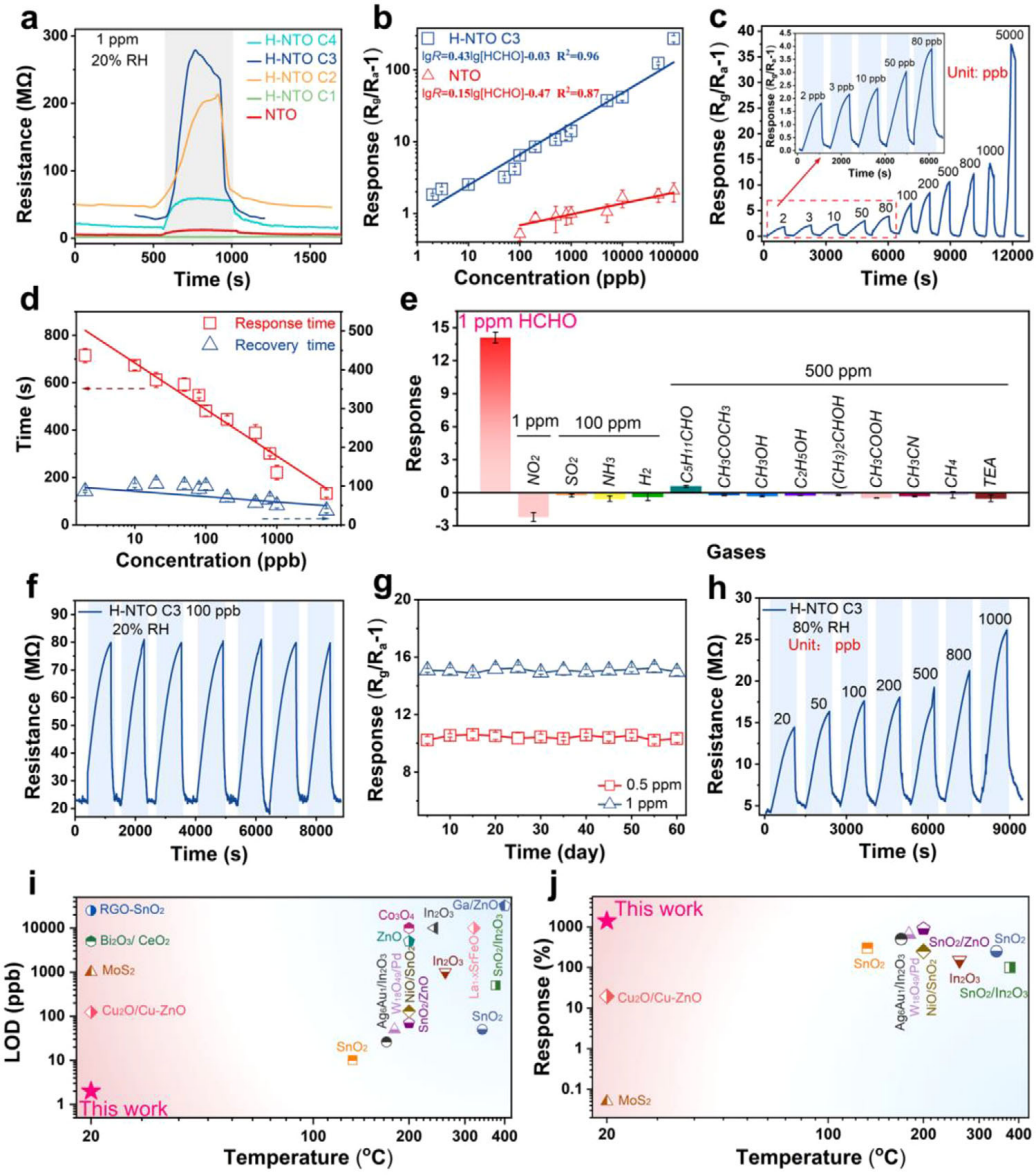
Gas sensing performance toward formaldehyde. a) Real‐time resistance changes of NTO and H‐NTO (C1‐C4) upon exposure to 1 ppm HCHO. b) Response values of NTO and H‐NTO C3 from 2 ppb to 100 ppm HCHO. c) Wide‐range sensing performance and d) response/recovery times of H‐NTO C3 vs HCHO concentration. e) Selectivity of H‐NTO C3 against reference gases. f) Cyclic stability under repeated HCHO exposure. g) Long‐term stability over 60 days. h) Wide‐range sensing at 80% RH. Comparison of i) detection limits and j) responses to 1 ppm HCHO of H‐NTO C3 with previously reported HCHO sensors.

**Figure** **3**a presents the response‐recovery profiles of pristine NTO and H‐NTO (C1‐C4) toward 1 ppm formaldehyde at 20 °C. While pristine NTO exhibits negligible resistance change, all H‐NTO C2‐C4 show pronounced resistance increases upon HCHO exposure, confirming the critical role of H⁺ exchange in enabling sensing activity. The response intensity improves progressively with increasing HCl concentration from 10^−4^ to 10^−3^ m, but declines with further increase of HCl concentration, likely due to structural degradation induced by excessive acid treatment. Figure S4 (Supporting Information) compares the sensing performance of H‐NTO samples (C1‐C4) toward 1 ppm HCHO. Among them, H‐NTO C3 exhibits the optimal performance, achieving a response value of 14.2 with response and recovery times of 128 and 85 s, respectively. Figure S5 (Supporting Information) shows that the baseline resistance of H‐NTO initially increases with rising H⁺ concentration up to 10^−2^ m but drops sharply when the H⁺ concentration reaches 10^−1^ m. A possible explanation is given in the Supporting Information.

Figure 3b–d highlight the wide‐range sensing performance of H‐NTO C3. The sensor exhibits a positive linear response across concentrations spanning 2 ppb to 100 ppm, following the relation lgR = 0.43 × lg[HCHO] (ppb) – 0.03. Importantly, the ultra‐low limit of detection (LOD) of 2 ppb not only surpasses pristine NTO and conventional semiconductor sensors but also enables reliable detection well below the WHO exposure limit of 80 ppb.^[^ ^22^ ^]^ H‐NTO C3 further demonstrates rapid, fully reversible response/recovery dynamics without the need for auxiliary heating or illumination (Figure 3d).

Excellent repeatability is confirmed by stable response cycles at 100 ppb HCHO (Figure 3f) and long‐term durability over two months, with only a 4% decrease in response (Figure 3g). Figure S6 (Supporting Information) shows that moderate temperature variations do not affect the H‐NTO sensor's response. The sensor also exhibits outstanding selectivity: negligible responses are observed for 100 ppm NH_3_, SO_2_, and H_2_, as well as for 500 ppm of common VOCs (Figure 3e; Figure S7, Supporting Information). For instance, the response to 500 ppm methanol is only 0.28, ≈50 times lower than that to 1 ppm HCHO. This remarkable selectivity ensures accurate HCHO detection in complex gas environments. Notably, the resistance change direction is independent of the redox properties of the analyte, distinguishing H‐NTO from conventional charge‐transfer‐based gas sensors. ^[^ ^33^, ^34^ ^]^ As shown in Figure S8 (Supporting Information), the sensing performance of H‐NTO does not rely on ambient oxygen. This characteristic provides a significant advantage over traditional metal‐oxide semiconductor sensors, which require oxygen to activate surface reactions and therefore cannot function properly in oxygen‐deficient conditions.

To elucidate the influence of ambient humidity on sensing performance, the response of H‐NTO C3 to formaldehyde was evaluated under various relative humidity (RH) levels. Typically, increasing RH deteriorates sensing performance because of the water poisoning effect.^[^ ^35^, ^36^ ^]^ Nevertheless, H‐NTO C3 still maintained high sensitivity and fast response/recovery dynamics for 20‐1000 ppb formaldehyde, even under 80% RH (Figure 3h; Figure S9, Supporting Information). Figure S10 (Supporting Information) presents the response curve of H‐NTO C3 to 1 ppm HCHO across a relative humidity range of 20‐80%, further demonstrating the excellent moisture resistance of the H‐NTO sensor.

Figure 3i,j, and Table S1 (Supporting Information) compare the performance of H‐NTO with recently reported formaldehyde sensors.^[^ ^8^, ^9^, ^11^, ^37^, ^38^, ^39^, ^40^, ^41^, ^42^, ^43^, ^44^, ^45^, ^46^, ^47^, ^48^, ^49^, ^50^, ^51^ ^]^ Most existing sensors require elevated operating temperatures, exhibit poor selectivity, and struggle to achieve ppb‐level detection. In sharp contrast, the H‐NTO sensor demonstrates an ultra‐low LOD of 2 ppb, exceptional selectivity over common VOCs such as methanol, acetone, and acetic acid, and a high response of 1400% toward 1 ppm formaldehyde at RT—far exceeding the performance of most reported counterparts. For example, the response of H‐NTO is nearly 28 000 times higher than that of MoS_2_,^[^ ^37^ ^]^ while its LOD is roughly three orders of magnitude lower. Similarly, SnO_2_ requires a high operating temperature of 330 °C,^[^ ^38^ ^]^ yet its response is <20% of that of H‐NTO and its LOD is 50‐fold higher. Beyond these advantages, the H‐NTO sensor also offers outstanding long‐term stability (>60 days) and strong humidity tolerance, underscoring its potential for reliable deployment in complex real‐world environments.

### 2.3 Possible Sensing Mechanism

**Figure 4:**
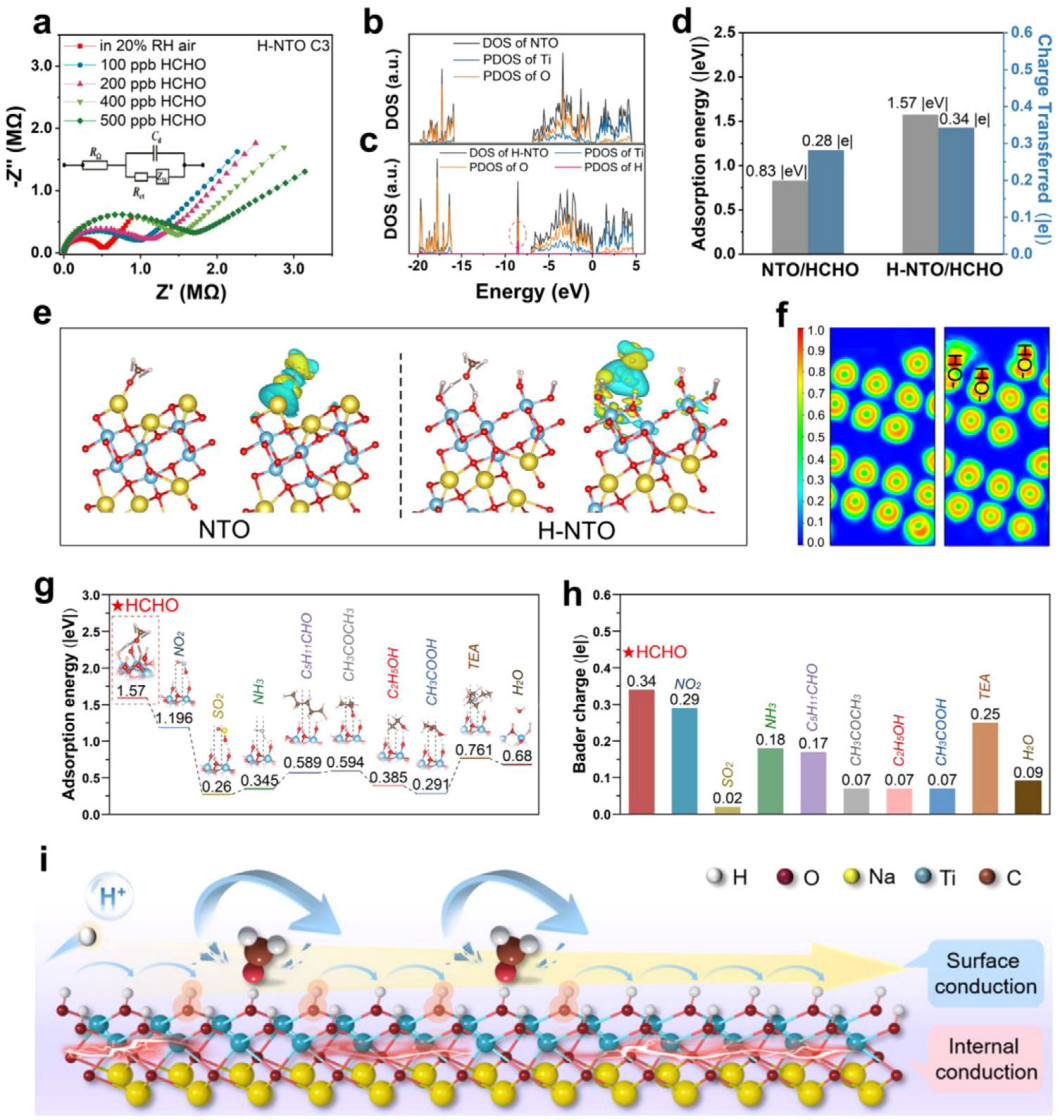
Mechanistic investigation of H‐NTO sensing behavior. a) Impedance spectra of H‐NTO C3 under 20% RH for 100–500 ppb HCHO. Density of states (DOS) of b) NTO and c) H‐NTO. d) Adsorption energy and charge transfer of HCHO on NTO and H‐NTO. e) Charge density difference maps. f) Electron localization function (ELF) maps. g) Adsorption energy diagrams of H‐NTO with different gas molecules. h) Bader charge distribution of H‐NTO upon gas adsorption. i) Schematic illustration of the sensing mechanism: surface hydroxyl groups selectively adsorb HCHO, inhibiting both surface‐proton and internal‐electron conduction.

The sensing mechanism of H‐NTO toward HCHO is fundamentally distinct from the oxygen ionosorption model of conventional MOS sensors. To elucidate the mechanism underlying the superior sensing performance, electrochemical impedance spectroscopy (EIS) was conducted over a frequency range of 10 Hz‐1 mHz. As shown in **Figure** **4**a, the Nyquist plots of H‐NTO consist of an inclined line at low frequencies and a semicircle at higher frequencies, features characteristic of proton conduction.^[^ ^52^ ^]^ The semicircle reflects the relaxation process of proton diffusion via hopping between adjacent hydroxyl groups, while the inclined line originates from diffusion‐related Warburg impedance.^[^ ^53^, ^54^ ^]^ Upon exposure to formaldehyde, the semicircle radius increases and the slope of the linear region decreases, evidencing strong suppression of proton conduction. This can be understood from the conductivity relation σ = σ_0_ exp(−E/RT),^[^ ^55^ ^]^ where formaldehyde adsorption both consumes hydroxyl sites and sterically blocks proton hopping, thereby raising the activation energy (E) and reducing proton conductivity. This proton conduction sensing mechanism also explains the temperature‐dependent sensing mechanism. As shown in Figure S6 (Supporting Information), operating temperature above 100  °C leads to a diminished response due to the thermal dehydration of surface hydroxyls.

The role of counterions (Na⁺ vs H⁺) was subsequently investigated. FTIR and XPS analyses confirm that —Ti─ONa in NTO is replaced by —Ti─OH in H‐NTO, producing abundant hydroxyl groups. From the perspective of molecular recognition, these hydroxyls act as hydrogen‐bond donors that preferentially interact with formaldehyde as a hydrogen‐bond acceptor.^[^ ^56^ ^]^ The redshift of hydroxyl groups observed in the FTIR spectra confirms the formation of hydrogen bonds during formaldehyde adsorption (Figure S11, Supporting Information).^[^ ^57^ ^]^ Density functional theory (DFT) calculations corroborate this: formaldehyde adsorption elongates the —Ti─O─H bond (1.16 → 1.20 Å) and alters bond angles (Figure S12, Supporting Information). Density of states (DOS) analysis (Figure 4b,c) shows that H⁺ incorporation narrows the bandgap near the Fermi level by compressing energy bands, lowering the excitation energy and aligning electronic levels with states favorable for adsorption. Hydrogen‐oxygen hybridization at deep energy levels further enriches surface oxygen electron density. Accordingly, the adsorption energy of HCHO on H‐NTO (1.57 |eV|) is nearly double that on NTO (0.83 |eV|, Figure 4d). Charge density maps (Figure 4e) confirm stronger electron transfer to HCHO on H‐NTO (0.34 |e| vs 0.28 |e|), validating the enhanced binding affinity.

To benchmark selectivity, the adsorption and charge transfer of eight additional gases with diverse functional groups were analyzed (Figure 4g,h; Table S2, Supporting Information). HCHO exhibited the highest adsorption energy (1.57 |eV|), whereas all other molecules remained below 1.2 |eV|, consistent with experimental selectivity. Differential charge density maps further reveal significant charge redistribution and bond strengthening for HCHO, in contrast to only minor perturbations for CH_3_COOH and SO_2_, reflecting weaker interactions and reduced adsorption stability. These findings highlight that adsorption energy and charge transfer fundamentally govern H‐NTO's high selectivity.

From the standpoint of transduction, H‐NTO exhibits faster response dynamics than NTO due to the smaller ionic radius of H⁺ and its Grotthuss‐type hopping through the hydroxyl network.^[^ ^52^, ^58^ ^]^ Because hydroxyl sites directly mediate HCHO adsorption, the activation energy for H⁺ transport is highly sensitive to formaldehyde. Electron localization function (ELF) analysis further clarifies the effect of H⁺ exchange. In NTO, nearly free electron (NFE) states are delocalized, supporting facile electron diffusion.^[^ ^59^ ^]^ In contrast, in H‐NTO, formaldehyde adsorption induces charge transfer with hydroxyl groups, forming potential wells that localize electrons, suppress NFE states, and restrict internal electronic conduction (Figure 4f).^[^ ^60^, ^61^ ^]^ As a result, both protonic and electronic pathways are simultaneously inhibited, producing sharp resistance changes and high sensitivity. Importantly, while humidity typically impairs gas sensing by competing for active sites,^[^ ^37^, ^62^ ^]^ water molecules in H‐NTO integrate into the proton‐conduction network and facilitate proton hopping, enabling the sensor to maintain strong formaldehyde sensitivity even under high‐RH conditions.

In conclusion, the sensing mechanism of H‐NTO is summarized in Figure 4i. Abundant hydroxyl groups provide specific recognition sites for formaldehyde, H⁺ hopping imparts fast and sensitive transduction, and adsorption suppresses both protonic and electronic conduction. Together, these synergistic effects account for the outstanding sensitivity, selectivity, and kinetics of the H‐NTO sensor.

### 2.4 Potential Application of H‐NTO‐Based Sensors

**Figure 5:**
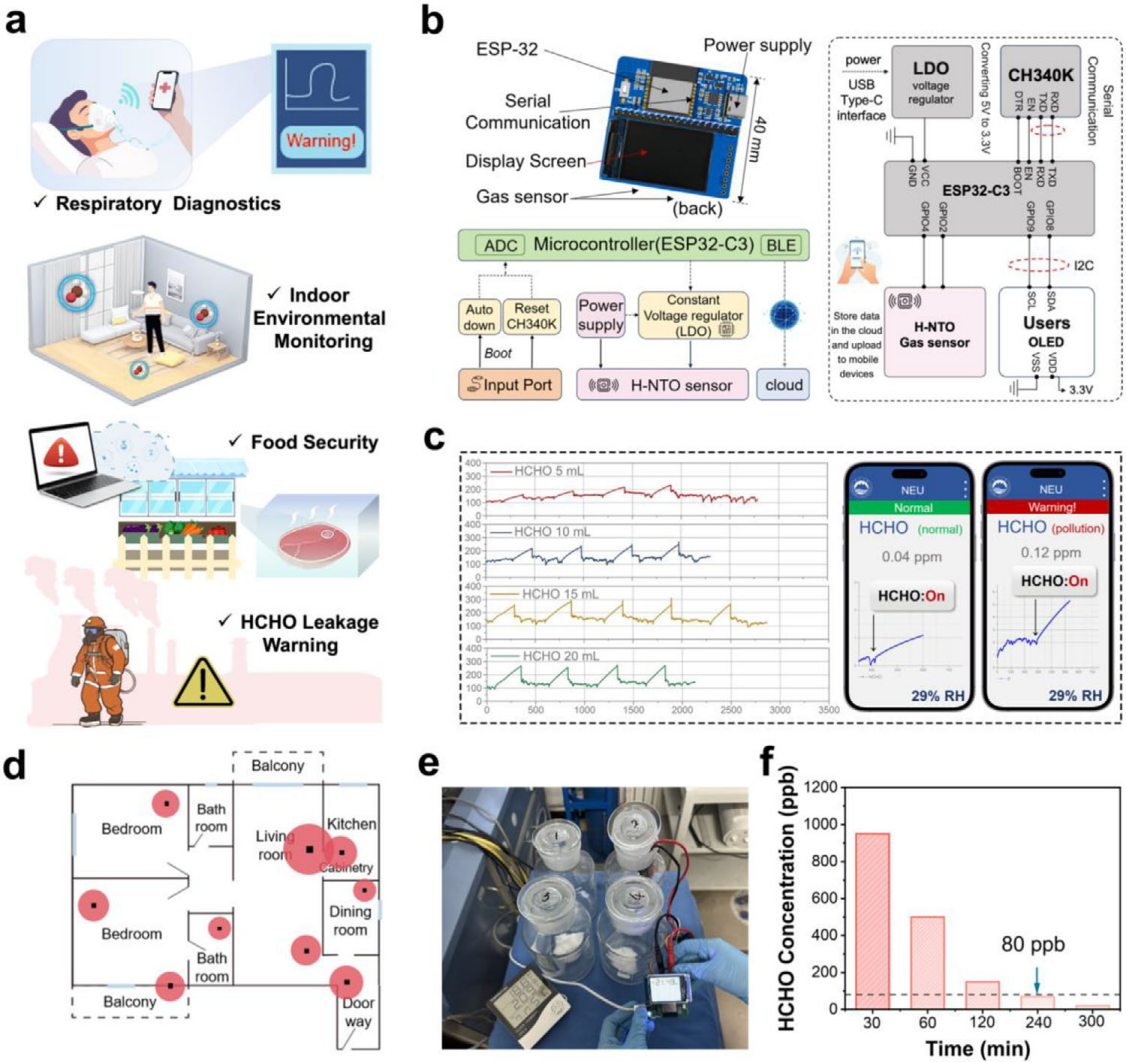
Smart monitoring and environmental application of H‐NTO sensor. a) Representative application scenarios. b) Schematic of the wireless HCHO monitoring and alarm system integrating ESP32 chip, H‐NTO sensor, power supply, and Wi‐Fi data transmission. c) Real‐time monitoring of emission sources and alarm notifications on mobile devices when exceeding safety thresholds. d) Indoor emission point distribution (red circles denote emission centers). e) Schematic of simulated emission sources. f) Variation of indoor HCHO concentration during simulated leakage.

Given the widespread presence of formaldehyde (HCHO) in industrial and domestic environments, and its recognition as a potential biomarker for breast cancer, HCHO sensors hold broad promise for applications in breath diagnostics, food safety, environmental monitoring, and industrial leak detection (**Figure** **5**a). To assess the practical utility of H‐NTO, we integrated the sensor into a 5 × 4 cm^2^ printed circuit board (PCB), constructing a wireless intelligent detection system (Figure 5b). The device incorporates the resistive H‐NTO sensor as the core element and connects to mobile devices via a Wi‐Fi module, enabling real‐time visualization and alerts through a dedicated application.

To emulate indoor HCHO emissions from wood‐based furniture, paints, and finishing materials, controlled release sources were prepared by applying varying volumes of HCHO solution (5–20 mL) onto cotton and sealing them in glass containers (Figure 5e). When deployed in rooms with these sources, the sensor produced immediate, distinct responses; once concentrations exceeded the 80 ppb safety threshold, a real‐time smartphone alert was triggered (Figure 5c). Stable baselines were recorded in rooms without HCHO, confirming excellent sensitivity. Importantly, the device maintained reliable performance under high humidity and in the presence of interfering vapors such as ethanol and acetic acid, demonstrating strong selectivity. Ventilation experiments further validated its practical relevance: ≈4 h of window opening was required to lower indoor HCHO levels below 80 ppb (Figure 5f).

**Figure 6:**
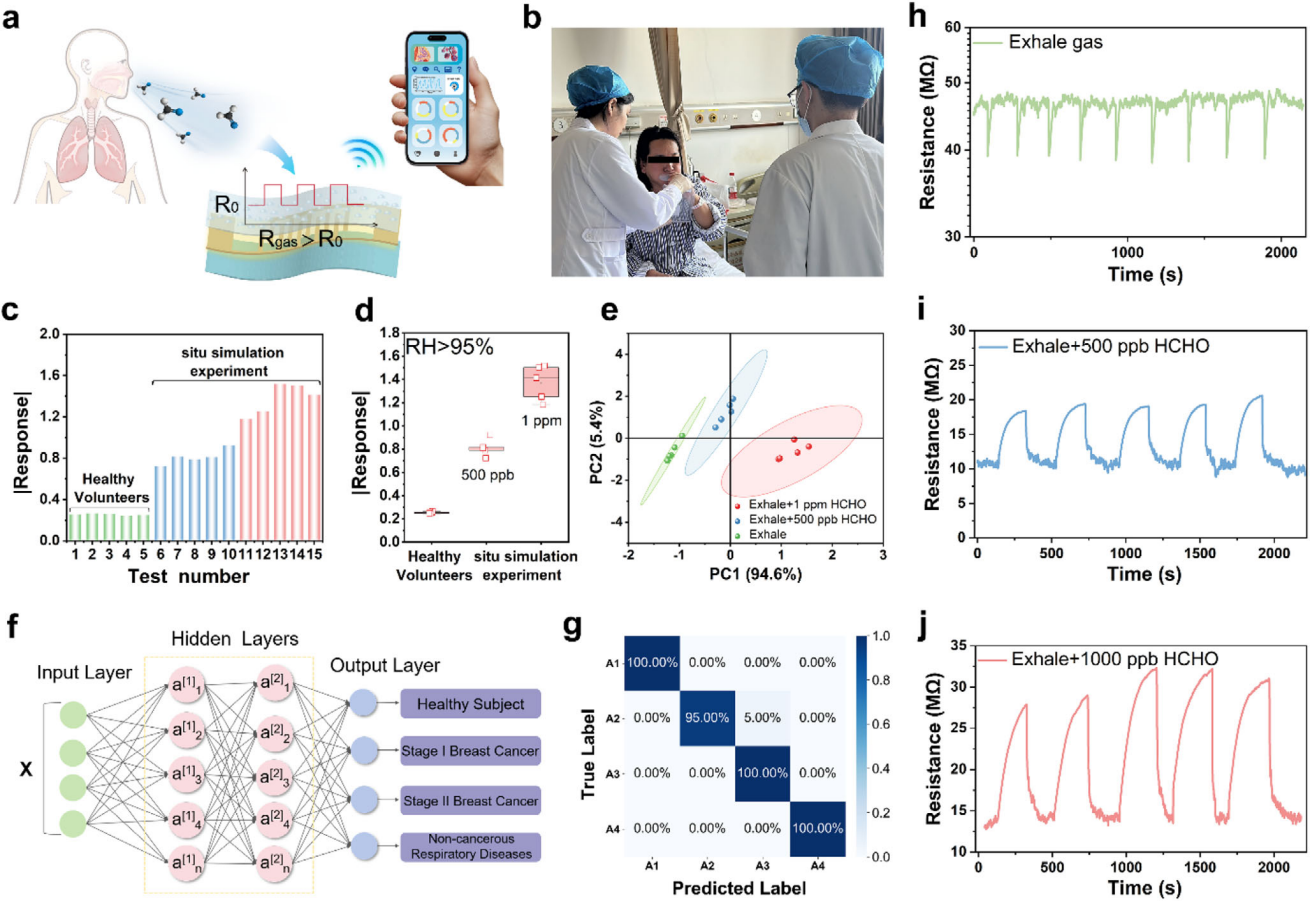
Breath analysis and diagnostic application of H‐NTO sensor. a) Schematic illustration of exhaled breath analysis. b) Collection of exhaled breath from breast cancer patients. c) Principal component eigenvalues of H‐NTO C3 responses to exhaled breath from healthy subjects (n = 5) and simulated breast cancer patients (n = 10). d) Box plot of sensor responses for both groups. e) Comparative analysis of exhaled gas composition. f) Schematic of the 1‐D CNN model architecture. g) Confusion matrix of breathing‐pattern classification: A1, normal (healthy); A2, mild abnormal (simulated stage I breast cancer); A3, pathological (stage II with lymph node metastasis); A4, non‐cancerous respiratory diseases. h) Response curve of healthy exhaled breath. Response curves after spiking with i) 500 ppb and j) 1 ppm formaldehyde.

Extending beyond environmental monitoring, H‐NTO shows strong potential for biomedical diagnostics. With ppb‐level detection and high selectivity, it was tested for non‐invasive breath analysis in breast cancer. Exhaled breath from healthy individuals and simulated patient samples (healthy breath spiked with HCHO) produced opposite sensor responses: resistance decreased in healthy breath but increased in simulated samples (**Figure** **6**h–j), enabling clear discrimination. Statistical analysis (Figure 6c,d) confirmed significantly higher responses for simulated patients, with compositional analysis (Figure 6e) showing no overlap between the two groups and a classification accuracy of 94.6%. Notably, breath samples from breast cancer patients (Figure 6b), collected following Section 4.6, induced resistance increases consistent with the simulated cases (Figure S13, Supporting Information), validating clinical relevance.

To further enhance recognition capability, a 1D convolutional neural network (1‐D CNN) was applied to classify sensor response patterns (Figure 6f; Equation S1, Supporting Information). From 100 datasets covering four breath state—(A1) normal breath, (A2) mild abnormal (stage I), (A3) pathological (stage II with lymph node metastasis), and (A4) non‐cancerous respiratory diseases (COPD)—the optimized CNN achieved an average classification accuracy of 99% (Figure 6g).

Collectively, these results demonstrate that H‐NTO, integrated with wireless circuitry and AI‐assisted analysis, enables highly selective, sensitive, and real‐time formaldehyde detection with broad application potential, ranging from indoor safety monitoring to early, non‐invasive diagnosis of breast cancer.

## 3 Conclusion

In conclusion, we introduce an innovative H⁺‐exchange strategy for highly selective, ppb‐level formaldehyde detection at room temperature. By replacing Na⁺ with H⁺ in NTO via dilute HCl treatment, the resulting H‐NTO exhibits outstanding sensing performance, including an ultralow LOD of 2 ppb, a wide detection range (2 ppb–100 ppm), high selectivity against common VOCs, fast recovery (85 s at 1 ppm HCHO), fully reversible response, and excellent stability over two months. Mechanistically, H⁺ exchange introduces abundant hydroxyl groups that act as specific adsorption sites for HCHO; adsorption increases the activation energy for proton hopping and creates charge‐transfer‐induced potential wells, thereby suppressing both surface proton conduction and internal electronic conduction. These synergistic effects underpin the exceptional selectivity and sensitivity of H‐NTO. Finally, integration into a wireless sensing platform enabled real‐time environmental monitoring and non‐invasive breath analysis, achieving 99% classification accuracy for breast cancer diagnosis with the aid of machine learning. This work establishes H‐NTO as a powerful material platform for next‐generation chemical sensors, bridging fundamental ion‐exchange chemistry with translational applications in health and environmental monitoring.

## 4 Experimental Section

### Synthesis of NTO

All chemicals were purchased and used directly in the experiments without further purification. NTO was synthesized following a previously reported method,^[^ ^26^, ^27^ ^]^ in which TiO_2_ powder (99%, Shanghai Chemical Technology Co., Ltd.) was used as a precursor and processed via a hydrothermal method (Figure 1a). Specifically, 0.1 g of TiO_2_ powder was mixed with 15 mL of 10 m NaOH solution and placed into a 20 mL stainless steel autoclave for a hydrothermal reaction at 140 °C for 36 h. After the reaction, the white suspension was separated by centrifugation and washed thoroughly with deionized water. The final solid product obtained was NTO.

### Synthesis of H‐NTO

H‐NTO was prepared by an ion‐exchange method.^[^ ^28^ ^.^ ^29^ ^]^ Specifically, NTO was dispersed in HCl solutions with concentrations ranging from 10^−1^ m to 10^−4^ m, and stirred for 10 min, and then centrifuged and washed with deionized water. The obtained samples were referred to as H‐NTO C1 to C4, respectively.

### Evaluation of Gas Sensing Performance

H‐NTO was mixed with ethanol and drop‐cast onto interdigitated electrodes on an alumina ceramic substrate (with an electrode gap of 20 µm), followed by drying in air. A DC voltage of 0.1 V was applied between the electrodes, and the gas sensing performance of NTO and H‐NTO was characterized using a gas sensing analysis system (CGS‐MT, Beijing Elite Tech Co.) equipped with a static gas distribution unit. During testing, the temperature was strictly controlled at 20  °C using a heating stage located beneath the sample. The RH was adjusted using a dynamic gas distribution system (DHD‐II, Beijing Gaoju Hi‐Tech Co.). VOC sample gases were prepared and controlled by evaporating VOC solutions and deionized water using a static injection method, and the amount of VOC solution used for evaporation was calculated using Equation S2 (Supporting Information). The response was defined as (*R*_gas_−*R*_air_)/*R*_air_, where *R*_gas_ and *R*_air_ represent the resistance of the sensor in HCHO and air, respectively. The response (recovery) time was defined as the time required for the resistance change to reach 90% of its maximum value after the injection (release) of HCHO.

### Characterization

XRD patterns were recorded using a PANalytical B.V. Empyrean diffractometer with Cu Kα radiation (λ = 1.5418 Å) in the 2θ range of 5–90°. The morphology of the NTO NRs and H‐NTO NRs was characterized by SEM (Hitachi SU‐8010) and TEM (JEOL JEM‐2100F). An XPS (Kratos XSAM‐800 spectrometer) with monochromatized Al Kα excitation was used to test the chemical states of the elements in the vacuum condition. The BET was acquired using Autosorb iQ Station (Quantachrome, America). FT‐IR spectroscopy (Bruker VERTEX 70) was employed to analyze the surface hydroxyl (—OH) groups on the H‐NTO NRs. EIS was obtained using a CHI 660E electrochemical workstation with an AC voltage of 0.1 V over a frequency range of 10 Hz to 1 mHz.

### Computational Details

All calculations were carried out for the material in the framework of DFT using the Vienna Ab initio Simulation Package (VASP 6.3.2).^[^ ^63^, ^64^ ^]^ The generalized gradient approximation (GGA) of the Perdew–Burke–Ernzerhof (PBE) function was used to describe the exchange‐correlation energy.^[^ ^65^ ^]^ The projected augmented wave (PAW) method and pseudopotentials were used to describe the interactions between valence electrons and ions.^[^ ^66^ ^]^ To ensure the efficiency of the computational results and parallel computing. A 2 × 2 × 1 k‐point grid under Monkhorst‐Pack is used in the optimization process, and 450 V truncation energy is set. The lattice parameters and ionic positions of all crystals were fully relaxed, and the convergence criteria for the total energy of all relaxed atoms and the final force were 10^−5^ eV and 0.05 eV/Å, respectively. The adsorption energy calculation formula is given by Equation S3 (Supporting Information).

### Exhaled Breath Collection from Breast Cancer Patients

Exhaled breath samples from subjects were collected using standardized polytetrafluoroethylene (PTFE) gas sampling bags. The detailed procedure was as follows: each sampling bag was equipped with an inlet and an outlet valve. After opening the valve, a silicone tube was used to connect the inlet of the gas sampling bag to a gas sampling pump at one end and to a disposable mouthpiece at the other. Subjects were instructed to exhale through the mouthpiece, and upon completion of breath collection, the valve was promptly closed to seal the sample.

### Ethical Approval

The study has been approved by the Ethics Committee of the First Affiliated Hospital of China Medical University for Medical Scientific Research (Approval No.~AF‐SOP‐07‐1.2‐01). This study was already registered in the National Health Security Information Platform Medical Research Registration and Filing Information System. All participants provided written informed consent before participating in the study.

### Statistical Analysis

For each type of sample, more than 10 independent sensors were fabricated, and each sensor was tested at least 10 times under each experimental condition to ensure the reliability and reproducibility of the statistical results. All experimental data are presented as mean ± standard deviation (SD). During data processing, the curves were first fitted and key features were extracted. Subsequently, principal component analysis (PCA) was employed to reduce the multidimensional features to 2D for data visualization. Data processing, feature extraction, and data visualization were performed using OriginPro 2023, MATLAB, Python, Vscode and Visio.

## Conflict of Interest

The authors declare no conflict of interest.

## Supporting information



Supporting Information

## Data Availability

The data that support the findings of this study are available from the corresponding author upon reasonable request.
