# Supplementary material for: H⁺ Exchange‐Driven ppb‐Level and High‐Selective Formaldehyde Detection at Room Temperature for Environmental and Clinical Applications
Source: Adv Sci (Weinh). 2025 Dec 8;13(11):e18324. doi: 10.1002/advs.202518324 (PMC12931182; doi:10.1002/advs.202518324)
Supplement: Supplementary file 1 — Supporting Information [file ADVS-13-e18324-s001.docx]

Supporting Information

H⁺ Exchange-Driven ppb-Level and High-Selective Formaldehyde Detection at Room Temperature for Environmental and Clinical Applications

Lubing Cai, Mengyang Pang, Zhaosong Liu, Yanfei Li, Jiani Li, Zhaorui Zhang, Fengshuang Zheng, Chao Li, Ang Zheng*, and Xuemin Zhang*

**Supplementary Figures**


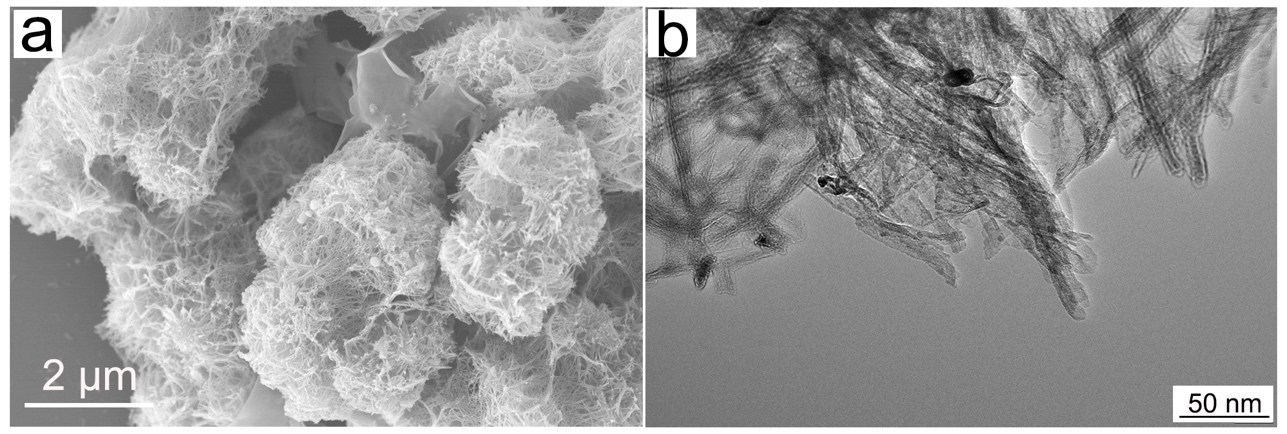


**Figure S1.** (a) SEM image and TEM image (b) of H-NTO C1.


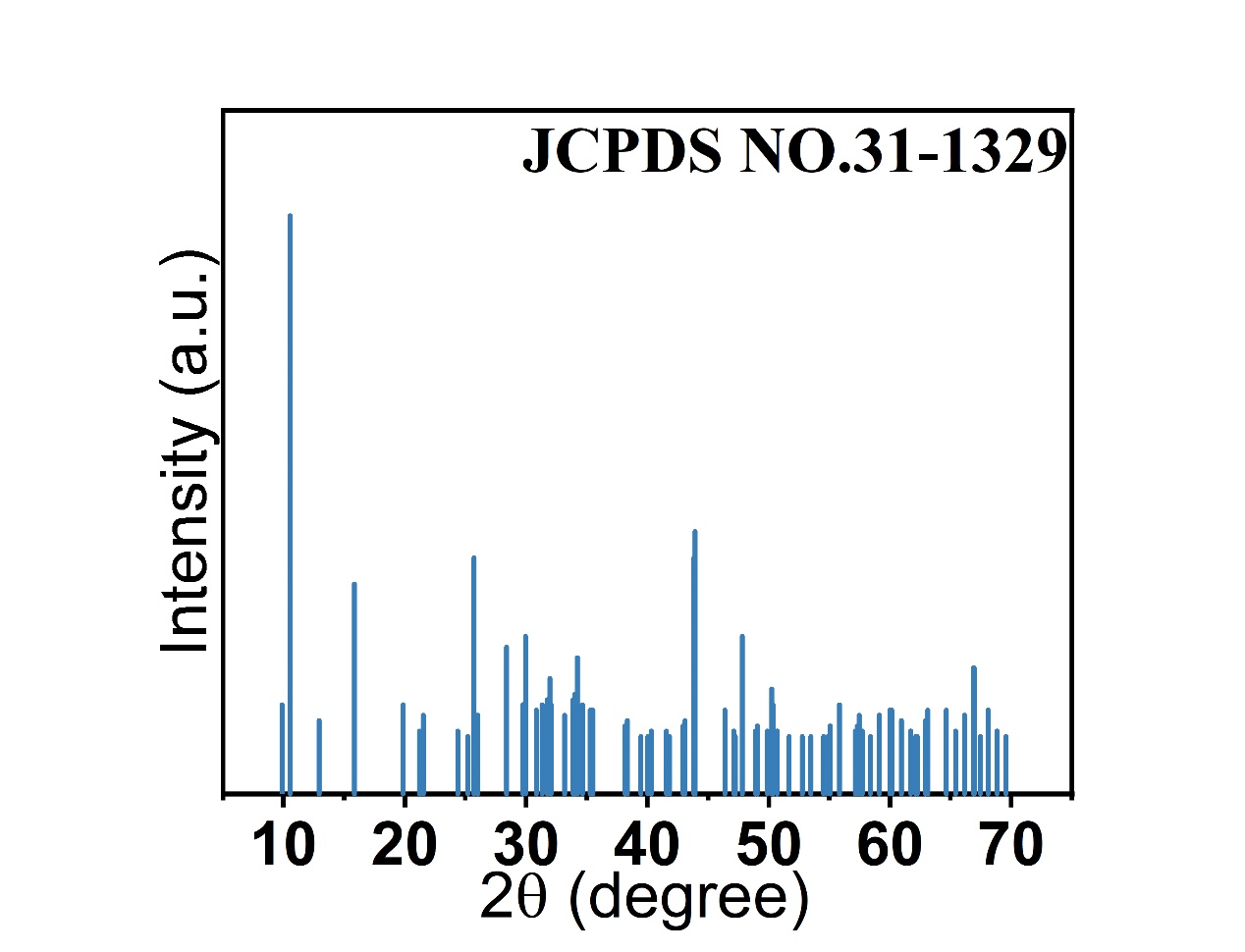


**Figure S2.** NTO NRs' XRD standard spectrum.


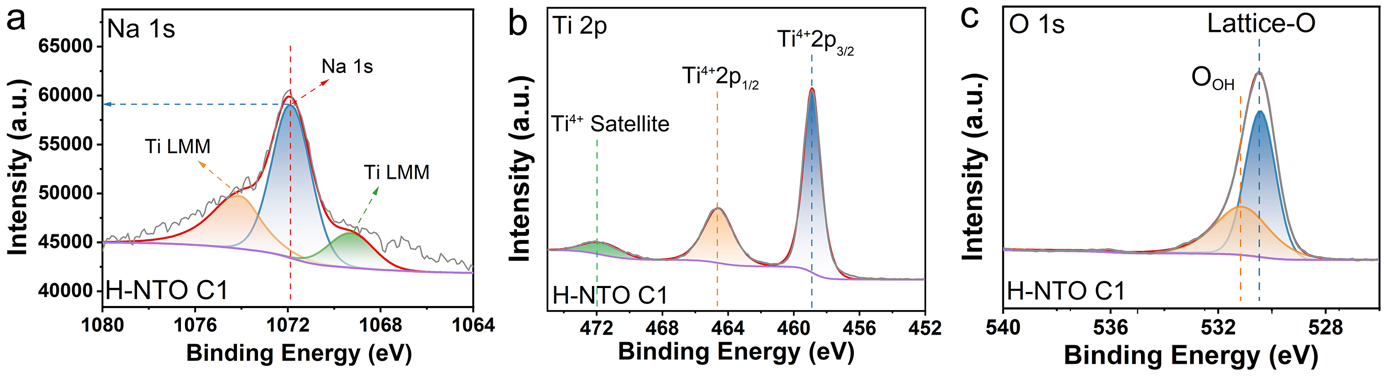


**Figure S3.** High-resolution XPS spectra of H-NTO C1: (a) Na 1s, (b) Ti 2p, and (c) O 1s.


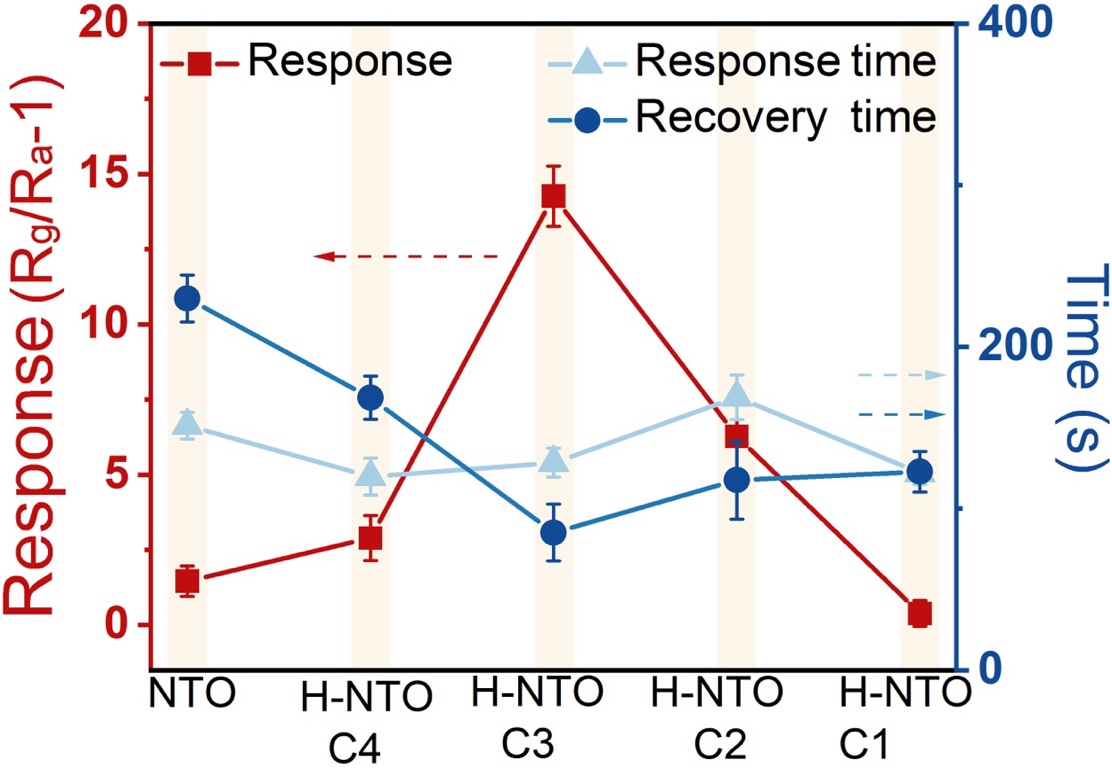


**Figure S4.** Comparison of performance of NTO and H-NTO toward 1 ppm HCHO.


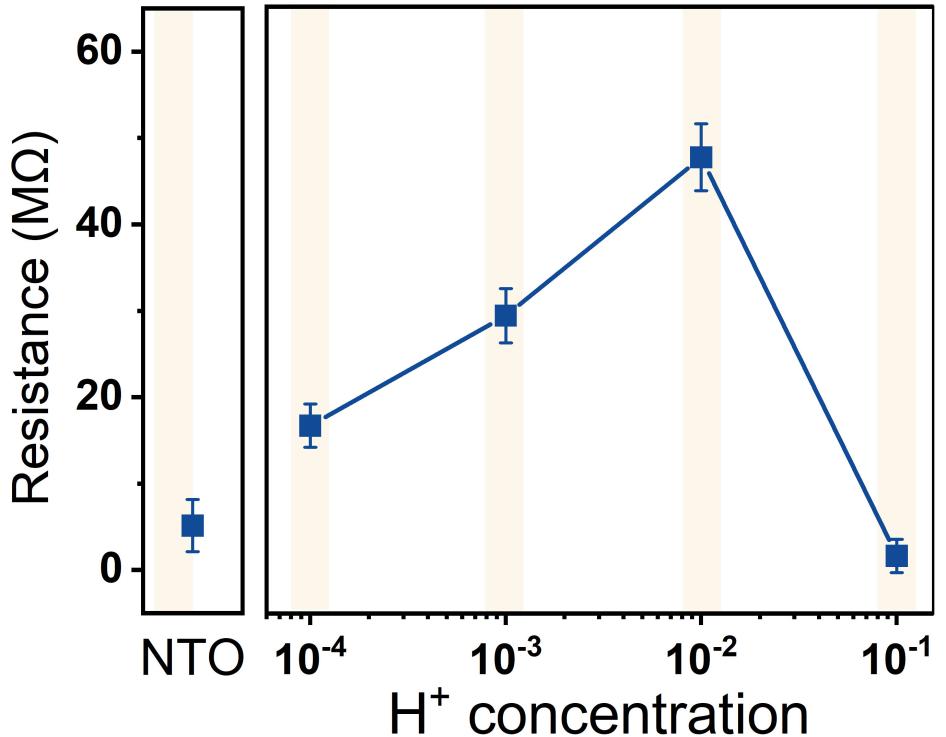


**Figure S5.** Plot of H-NTO resistance versus H⁺ concentration.

As Figure S5 shown, the resistance of H-NTO initially increases with rising H⁺ concentration up to 10^-2^ M but drops sharply when the concentration reaches 10^-1^ M. This behavior can be explained by ion conduction. According to previous studies, the conductivity (σ) of an ion-conducting material is determined by the ion concentration (n) and mobility (μ):

$$\sigma=ne\mu$$

where *e* is the elementary charge. During the H⁺ exchange process, Ti–ONa groups in NTO are converted into Ti–OH in H-NTO. Since Ti–OH is a weak acid, the number of dissociable ions on the H-NTO surface is initially lower than in NTO, leading to an increase in resistance with H⁺ concentration. However, Ti–OH groups also serve as active sites for proton hopping. When their density becomes sufficient, proton mobility increases due to reduced activation energy for surface transport, thereby enhancing conductivity (as observed from H-NTO C2 to C1). Furthermore, excessive H⁺ exchange partially collapses the layered structure, increasing inter-ribbon contact area and further affecting conductivity. These results demonstrate that acid concentration strongly influences the electrical properties of H-NTO.


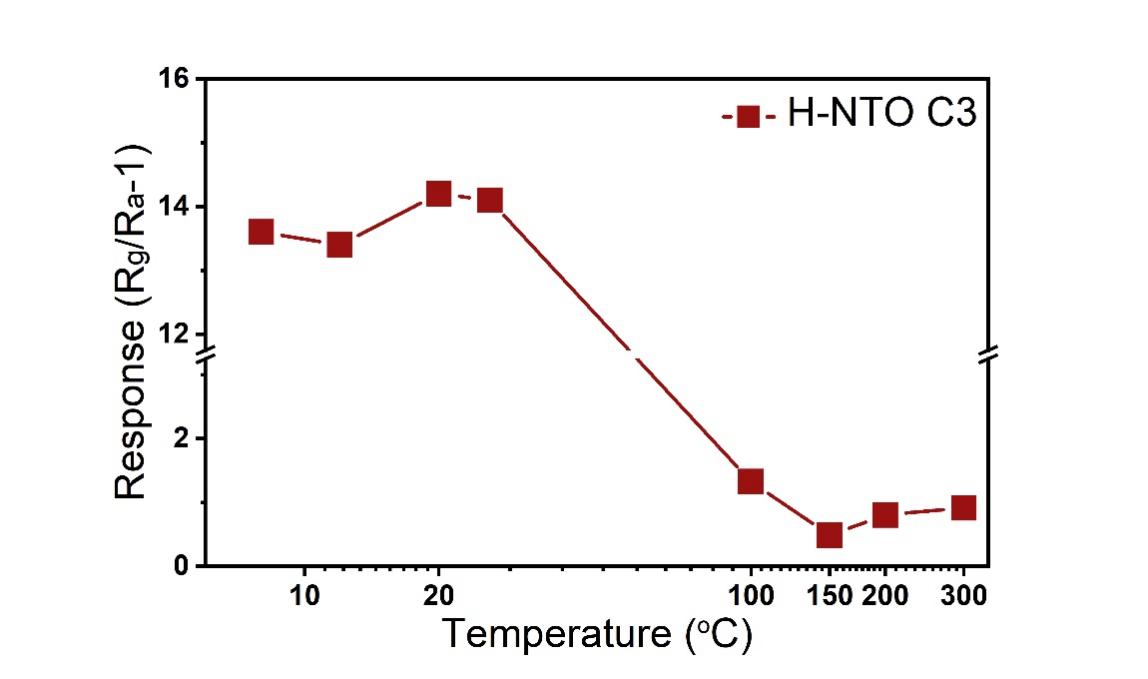


**Figure S6.** Response of H-NTO C3 to 1 ppm HCHO at different temperatures.


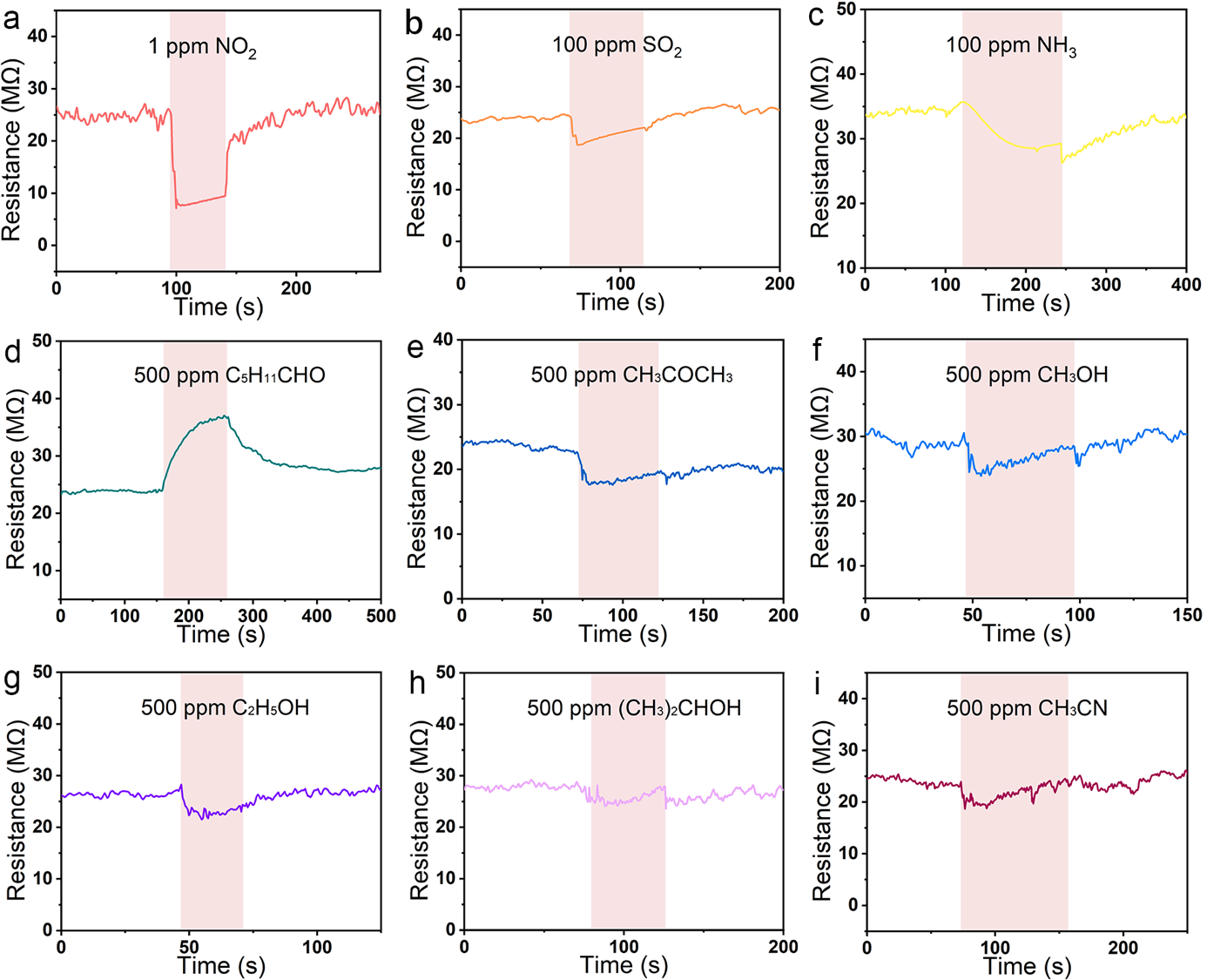


**Figure S7.** Real-time resistance changes when H-NTO C3 are exposed to NO_2_ (a), SO_2_ (b), NH_3_ (c), C_5_H_11_CHO (d), CH_3_COCH_3_ (e), CH_3_OH (f), C_2_H_5_OH (g), (CH_3_)_2_CHOH (h), CH_3_CN (i).


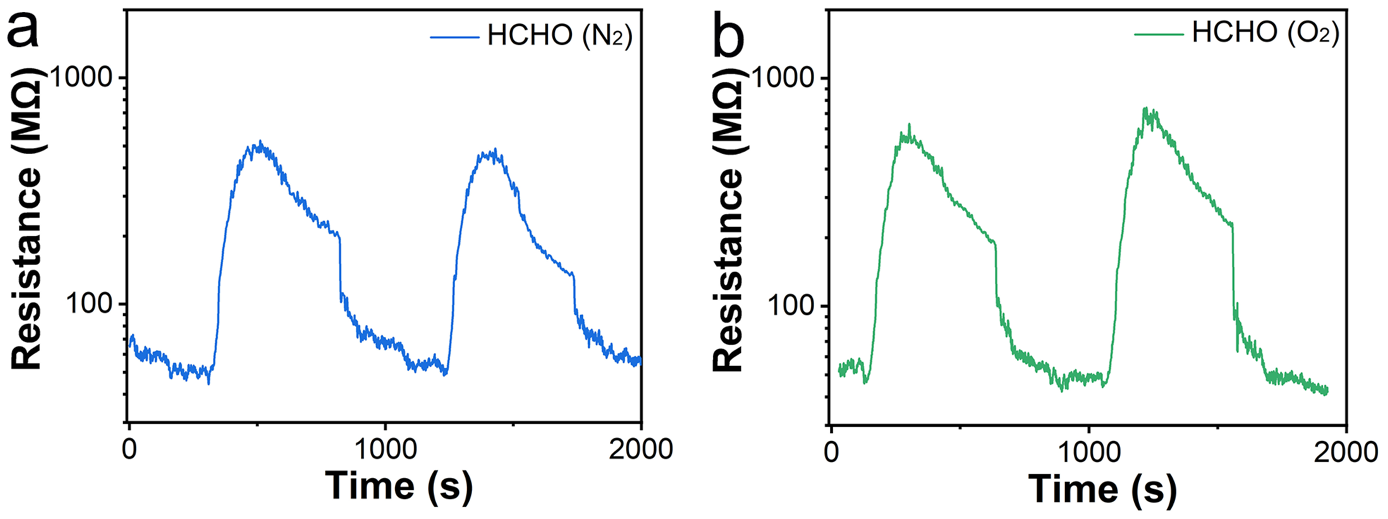


**Figure S8.** Response curves of H-NTO toward HCHO under oxygen-deficient (a) and oxygen-rich conditions (b).


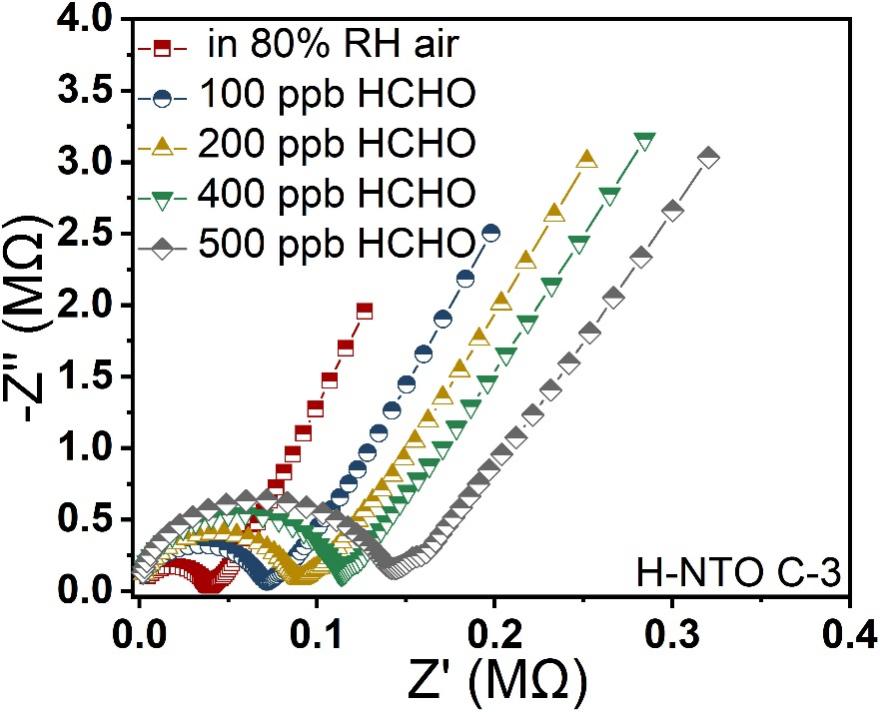


**Figure S9.** Impedance spectra of H-NTO C3 at RH 80% for 100-500 ppb HCHO.


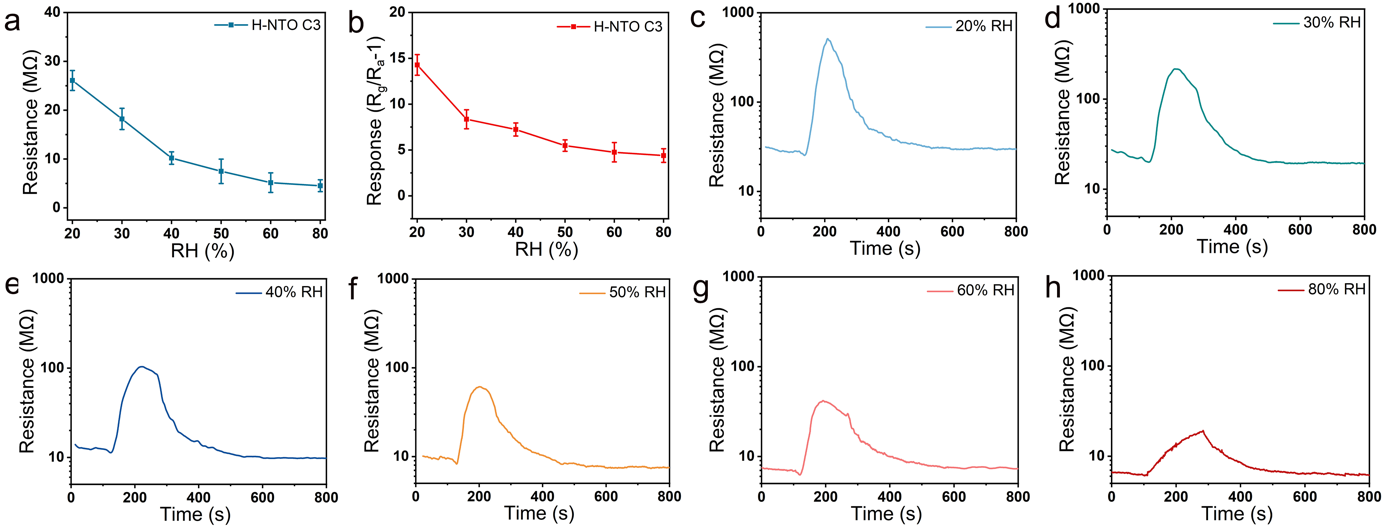


**Figure S10.** The base-resistance (a) and response (b) of H-NTO C3 sensor at varying humidity levels. (c-h) Response curves of H-NTO C3 to 1ppm HCHO at varying humidity levels.


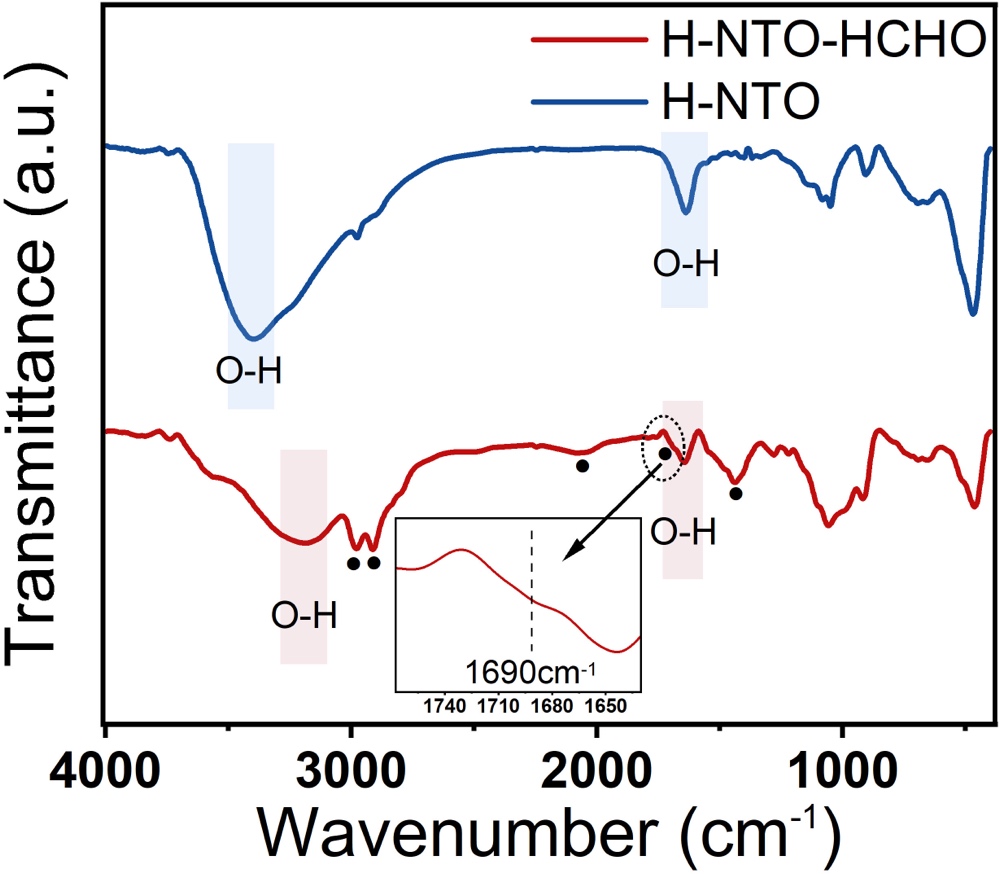


**Figure S11.** FTIR spectra of H-NTO C3 before and after adsorption of formaldehyde.


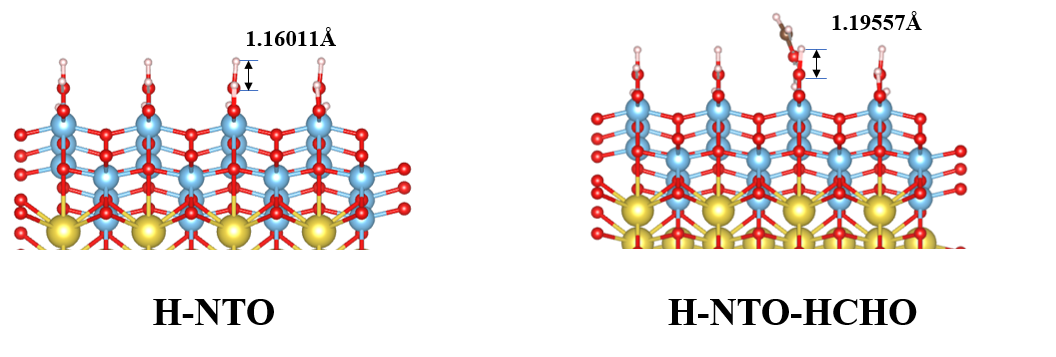


**Figure S12.** Hydrogen bond length variation of H-NTO after formaldehyde adsorption.


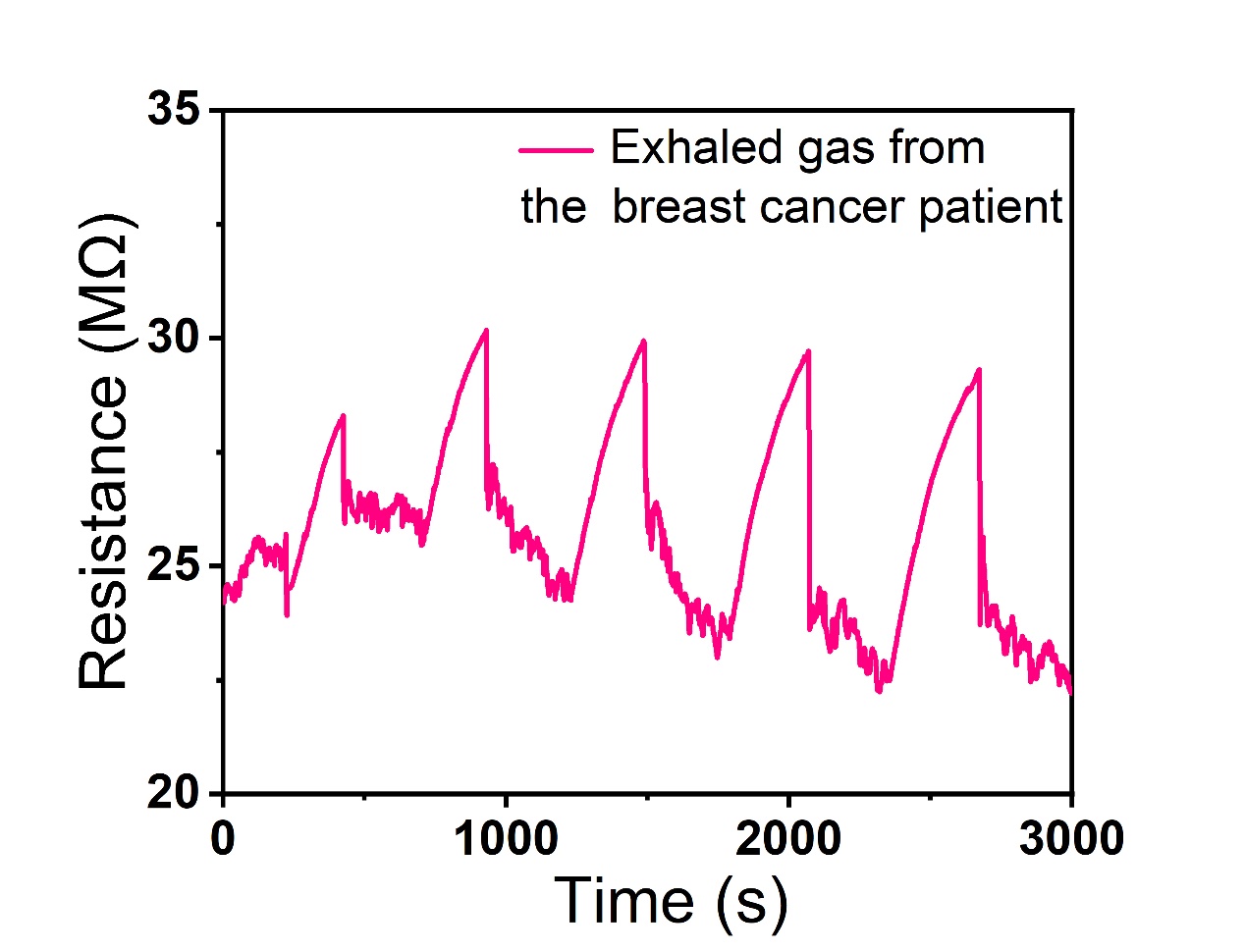


**Figure S13.** Real-time resistance changes testing of exhaled gas in breast cancer patients.

Exhaled breath samples from subjects were collected using standardized polytetrafluoroethylene (PTFE) gas sampling bags. The detailed procedure was as follows: each sampling bag was equipped with an inlet and an outlet valve. After opening the valve, a silicone tube was used to connect the inlet of the gas sampling bag to a gas sampling pump at one end and to a disposable mouthpiece at the other. Subjects were instructed to exhale through the mouthpiece, and upon completion of breath collection, the valve was promptly closed to seal the sample. This study was already registered in the National Health Security Information Platform Medical Research Registration and Filing Information System. All participants provided written informed consent before participating in the study. The approval number is Approval No.~AF-SOP-07-1.2-01.

$\overline{x_{i}}$*=*$\frac{x_{i}-\mu}{\sigma}$ (S1)

**Equation S1.** Where $\overline{x_{i}}$ and *xi* are the normalized and original signal values of each batch, respectively, while *μ* and *σ* express the mean and standard deviation, respectively.^[1]^

$$C_{ppm}=\frac{22.4\rho\cdot T{\cdot V}_{1}}{273M\cdot V}\times1000 (S2)$$

**Equation S2.** VOC gas concentration calculation equation.^[2]^ The required volume of organic solvents was injected onto the evaporation platform via a micro syringe, and the evaporation table controlled the evaporation temperature of the organic solvents in the test chamber allowing them to fully evaporate into the gas phase. The concentration of the analyte gas was calculated by Equation S3, where *C_ppm_* is the concentration of the organic solvents (ppm), *ρ* is the density of anhydrous liquid organic solvents (g·mL^-1^), *T* is the testing temperature (298 K), *V_1_* is the volume of organic solvents (μL), *M* is the molecular weight of organic solvent (g·mol^-1^) and *V* is fixed chamber volume (5 L).

$E_{\mathrm{ads}}=E_{Composite structure}-E_{Adsorption surface}-E_{\mathrm{adsorbate}}$ (S3)

**Equation S3.** The adsorption energy formula.

**Supplementary Table**

**Table S1.** Sensing performance of recently reported electrical HCHO sensors.

| Sensing layer | Response | Working temperature | Detection range | Long-term stability | Ref. |
| --- | --- | --- | --- | --- | --- |
| In_2_O_3_ | 2.9 at 10 ppm | 240 ^o^C | 10-200 ppm | 30 days | 3 |
| SnO_2_ | 3 at 1 ppm | 133 ^o^C | 0.01-100 ppm | 60 days | 4 |
| ZnO/SnO_2_ | 9 at 1 ppm | 200 ^o^C | 0.5-200 ppm | 30 days | 5 |
| W_18_O_49_/Pd-2 | 6.5 at 1 ppm | 180 ^o^C | 50 ppb-10 ppm | 30 days | 6 |
| Ag_6_Au_1_/In_2_O_3_ | 5 at 1 ppm | 170 ^o^C | 50 ppb-5 ppm | 15 days | 7 |
| 1T/2H MoS_2_ | 0.05% at 1 ppm | RT | 1-500 ppm | / | 8 |
| Bi_2_O_3_/CeO_2_ | 0.57 at 20 ppm | RT | 5-100 ppm | 20 days | 9 |
| rGO/TiO_2_ | 0.4% at 0.5 ppm | RT | 0.1-0.5 ppm | 140 days | 10 |
| InOCl | 1.5 at 1 ppm | 200 ^o^C | 1-50 ppm | 16 days | 11 |
| La_1-x_Sr_x_FeO_3_ | 1 at 10 ppm | 320 ^o^C | 10-50 ppm | 30 days | 12 |
| graphene | 0.05 at 5 ppm | RT | 5-150 ppm | 60 days | 13 |
| Ti_3_C_2_T_x_/SnO_2_ | 0.15 at 1 ppm | RT | 0.5-100 ppm | 30 days | 14 |
| Co-doped TiO_2_ | 19 at 1 ppm | 86 ^o^C | 250 ppb-100 ppm | 16 days | 15 |
| RGO-SnO_2_ | 23 at 25 ppm | RT | 25-200 ppm | / | 16 |
| Ni−In_2_O_3_/WS_2_ | 0.17 at 2 ppm | RT | 0.05-20 ppm | 60 days | 17 |
| α-Fe_2_O_3_ | 0.28 at 50 ppm | 325 ^o^C | 50-200 ppm | / | 18 |
| **H-NTO NRs** | **14.2 at 1ppm** | **RT** | **2 ppb-100 ppm** | **60 days** | **This Work** |

**Table S2.** Adsorption energies and charge transferred between different molecules and H-NTO.

| Composite structure | Adsorption energy/eV | Charge transferred/e^-^ |
| --- | --- | --- |
| H-NTO-SO_2_ | -0.26 | -0.02 |
| H-NTO-NO_2_ | -1.196 | -0.29 |
| H-NTO-NH_3_ | -0.345 | -0.18 |
| H-NTO-CH_3_COOH | -0.291 | -0.07 |
| H-NTO-C_2_H_5_OH | -0.385 | -0.07 |
| H-NTO-CH_3_COCH_3_ | -0.594 | -0.07 |
| H-NTO-C_5_H_11_CHO | -0.589 | -0.17 |
| H-NTO-TEA | -0.761 | -0.25 |

**References**

1. Q. Zhou, Q. Ding, Z. Geng, C. Hu, L. Yang, Z. Kan, B. Dong, M. Won, H. Song, L. Xu, A Flexible Smart Healthcare Platform Conjugated with Artificial Epidermis Assembled by Three‑Dimensionally Conductive MOF Network for Gas and Pressure Sensing, *Nano-Micro Lett.* **2024**, *17*, 50.
2. Y. Zhang, C. Zhang, Z. Zhang, H. Zong, P. Tan, L. Luo, Y. Luo, G. Duan, Double-Phase Ga-Doped In_2_O_3_ Nanospheres and Their Self-Assembled Monolayer Film for Ultrasensitive HCHO MEMS Gas Sensors, *Small* **2025**, *21*, 2411422.
3. X. Huang, Z. Tang, Z. Tan, S. Sheng, Q. Zhao, Hierarchical In_2_O_3_ nanostructures for improved formaldehyde: sensing performance, *J. Mater. Sci.: Mater. Electron* **2021**, *32*, 11857.
4. X. Ma, R. Gao, T. Zhang, X. Sun, T. Li, S. Gao, X. Zhang, Y. Xu, X. Cheng, L. Huo, Mesoporous SnO_2_ nanospheres sensor for fast detection of HCHO and its application in safety detection of aquatic products, *Sens. Actuators, B* **2023**, *374*, 132844.
5. C. Lou, C. Yang, W. Zheng, X. Liu, J. Zhang, Atomic layer deposition of ZnO on SnO_2_ nanospheres for enhanced formaldehyde detection, *Sens. Actuators, B* **2021**, *329*, 129218.
6. P. Wang, S. Guo, Z. Hu, T. Li, S. Pu, H. Mao, H. Cai, Z. Zhu, H.-Y. Li, H. Liu, W_18_O_49_ sensitized with Pd nanoparticles for ultrasensitive ppb-level formaldehyde detection, *Chem. Eng. J.* **2023**, *456*, 140988.
7. J. Huang, J. Li, Z. Zhang, J. Li, X. Cao, J. Tang, X. Li, Y. Geng, J. Wang, Y. Du, Bimetal Ag NP and Au NC modified In_2_O_3_ for ultra-sensitive detection of ppb-level HCHO, *Sens. Actuators, B* **2022**, *373*, 132664.
8. Z. Wang, X. Zhao, F. Hong, H. Wang, H. Lei, Z. Chou, J. Zheng, Room-Temperature, Flexible Formaldehyde Gas Sensors Using Titanium-Incorporated 1T/2H MoS_2_, *ACS Appl. Mater. Interfaces* **2024**, *16*, 65185.
9. X. Meng, S. Kang, Z. Zhao, G. Jin, Z. Shao, L. Wu, Core-shell Bi_2_O_3_/CeO_2_ heterojunction for enhanced formaldehyde gas sensor, *Ceram. Int.* **2025**, *51*, 6067.
10. Z. Ye, H. Tai, T. Xie, Z. Yuan, C. Liu, Y. Jiang, Room temperature formaldehyde sensor with enhanced performance based on reduced graphene oxide/titanium dioxide, *Sens. Actuators, B* **2016**, *223*, 149.
11. Y. Bian, L. Nie, A. Wang, L. Zhang, R. Yue, N. Han, Y. Chen, Facile synthesis of stoichiometric InOCl mesoporous material for high performance formaldehyde gas sensors, *Sens. Actuators, B* **2020**, *319*, 128078.
12. P.-J. Yao, J. Wang, W.-L. Chu, Y.-W. Hao, Preparation and characterization of La_1-x_Sr_x_FeO_3_ materials and their formaldehyde gas-sensing properties, *J. Mater. Sci.* **2012**, *48*, 441.
13. J. Fan, H. Li, H. Hu, Y. Niu, R. Hao, A. Umar, M. S. Al-Assiri, M. A. Alsaiari, Y. Wang, An insight into improvement of room temperature formaldehyde sensitivity for graphene-based gas sensors, *Microchem. J.* **2021**, *160*, 105607.
14. Y. Zhang, M.-Y. Wang, X.-G. San, Y.-B. Shen, G.-S. Wang, L. Zhang, D. Meng, Ti_3_C_2_Tx/SnO_2_ P-N heterostructure construction boosts room temperature detecting formaldehyde, *Rare Metals* **2023**, *43*, 267.
15. Q. Rong, Y. Li, S. Hao, S. Cai, C. Wolverton, V. P. Dravid, T. Zhai, Q. Liu, Raspberry-like mesoporous Co-doped TiO_2_ nanospheres for a high-performance formaldehyde gas sensor†, *J. Mater. Chem. A* **2021**, *9*, 6529.
16. B. Manna, S. Acharyya, I. Chakrabarti, P. K. Guha, Graphene Oxide Wrapped Hollow SnO_2_ Sphere for Room Temperature Formaldehyde Sensing: An Insight Through Computational Analysis & Experimental Study, *IEEE Trans. Electron Devices* **2020**, *67*, 3767.
17. D. Zhang, Y. Cao, Z. Yang, J. Wu, Nanoheterostructure Construction and DFT Study of Ni-Doped In_2_O_3_ Nanocubes/WS_2_ Hexagon Nanosheets for Formaldehyde Sensing at Room Temperature, *ACS Appl. Mater. Interfaces* **2020**, *12*, 11979.
18. P. Das, B. Mondal, K. Mukherjee, Facile synthesis of pseudo-peanut shaped hematite iron oxide nano-particles and their promising ethanol and formaldehyde sensing characteristics, *RSC Adv.* **2014**, *4*, 31879.
